# A Bread Wheat Line with the Substituted Wild Emmer Chromosome 4A Results in Fragment Deletions of Chromosome 4B and Weak Plants

**DOI:** 10.3390/plants14071134

**Published:** 2025-04-05

**Authors:** Yu Qiu, Fei Lu, Bohao Yang, Xin Hu, Yanhao Zhao, Mingquan Ding, Lei Yang, Junkang Rong

**Affiliations:** 1 College of Advanced Agricultural Sciences, Zhejiang A&F University, Hangzhou 311300, China; qy13548148573@163.com (Y.Q.); irislf0726@163.com (F.L.); b19550185866@163.com (B.Y.); huxin98@foxmail.com (X.H.); junkangrong@126.com (J.R.); 2 Institute of Future Agriculture, Northwest A&F University, Yangling 712100, China; 3 Tonglu County Agricultural Technology Extension Centre, Hangzhou 311500, China; 18357173189@163.com

**Keywords:** wild emmer wheat, weak plant, chromosome arm substitution line, chromosomal fragment deletion

## Abstract

In response to the growing genetic uniformity within wheat populations, developing efficient wheat–alien translocation strategies has become critically important. We observed that several offspring of the common wheat (*Triticum aestivum* L.)–wild emmer (*Triticum turgidum* L. var. *dicoccoides*) chromosome arm substitution line (CASL4AL) exhibited stunted growth, including significantly reduced plant height, spike length, spikelet number, and stem width compared to normal plants. Integrative transcriptomic analyses (RNA-Seq and BSR-Seq) revealed a statistically significant depletion (*p* < 0.01) of single nucleotide polymorphisms (SNPs) on chromosome 4B in compromised plants. Chromosome association analysis of differentially expressed genes (DEGs, up- or downregulated) revealed that downregulated genes were predominantly located on chromosome 4B. The 1244 downregulated DEGs on Chr4B were employed for Gene Ontology (GO) and Kyoto Encyclopedia of Genes and Genomes (KEGG) analyses, and RNA metabolic processes, DNA repair, and transport systems were significantly enriched by GO analysis; however, only the mRNA surveillance pathway was enriched by KEGG enrichment. Molecular marker profiling showed a complete absence of target amplification in the critical 0–155 Mb region of chromosome 4B in all weak plants. Pearson’s correlation coefficients confirmed significant associations (*p* < 0.01) between 4B-specific amplification and weak phenotypes. These results demonstrate that 4B segmental deletions drive weak phenotypes in CASL4AL progeny, and provide experimental evidence for chromosome deletions induced in wild emmer chromosome substitution lines. This study highlights the potential of wild emmer as a valuable tool for generating chromosomal variations in wheat breeding programs.

## 1. Introduction

Wheat (*Triticum aestivum* L.), cultivated on approximately 200 million hectares globally, is a pivotal crop in the global agricultural landscape, contributing at least 20% of calories and proteins to human diets [1,2,3]. However, the converging pressures of climate change and rapid population growth have dramatically increased demands for wheat production. Common wheat, characterized as a hexaploid crop (2n = 6x = 42, AABBDD), originated through two successive polyploidization events involving species from the genera *Triticum* and *Aegilops* [4]. Unfortunately, the repetitive cycles of polyploidization, combined with thousands of years of domestication, have caused a marked depletion of genetic diversity in present-day wheat cultivars [5,6]. Consequently, the primary gene pool of common wheat has been extensively exploited, resulting in significantly diminished breeding efficiency that threatens long-term yield stability [7]. Therefore, it is necessary to put urgent efforts into enhancing wheat breeding strategies.

Despite the low genetic diversity in common wheat, wild relatives, with their rich genetic variability and beneficial traits, offer valuable resources for wheat breeding [7]. The inherent genomic plasticity of polyploid crops facilitates the effective transfer of superior genes from wild relatives into wheat, serving as a crucial strategy for developing new varieties with enhanced resistance and yield potential. Notable successes include the widely utilized wheat–Rye (*Secale cereale* L.) 1BL·1RS translocation lines, which have introduced a series of genes of disease resistance *Lr26* (leaf rust resistance gene)/*Sr31* (stem rust resistance gene)/*Yr9* (stripe rust resistance gene)/*Pm8* (powdery mildew resistance gene) [8,9,10] into global wheat breeding programs. Similarly, there is the hybrid system of wheat—*Agropyron cristatum* developed through nearly 30 years of dedicated research by Li et al. [11,12]. This represents a significant milestone in the distant hybrid breeding of wheat. Nevertheless, the current pace of such breeding achievements remains insufficient to meet the demands of breeding new varieties amid rapidly changing climatic conditions.

Previous studies have indicated that wild relatives can serve as valuable sources for incorporating useful alien genes into common wheat. Some alien chromosomes are capable of inducing structural variations in wheat chromosomes. The gametocidal (Gc) system is one of the most utilized methods for creating translocation lines [13]. Gc chromosomes, identified in the *Aegilops* genus’s C, S, and M genomes, can induce chromosomal non-homoeologous translocations and deletions, either between common wheat chromosomes or between common wheat and alien chromosomes [14]. Endo et al. utilized Gc chromosomes to create a comprehensive set of chromosome deletion lines in the Chinese Spring background in 1996 [15]. Since then, more wild relatives have been identified as capable of inducing chromosomal mutations. For example, chromosome 5P from *Aegilops cristatum* can induce non-homoeologous chromosome translocations in common wheat and was found by Han et al. [16]. Three octoploid *Trititrigia* accessions (TE261-1, TE266-1, and TE346-1), found by Cui et al. to have chromosomal structural differences, were found on chromosomes 1A, 6A, 6B, 2D, and 7D [17].

Among the wild relatives of wheat, wild emmer (*Triticum turgidum* L. var. *dicoccoides*), as the tetraploid progenitor of common wheat, is recognized as a pivotal germplasm reservoir [18]. Wild emmer has evolved extensive genetic diversity through prolonged environmental adaptation, while developing valuable traits such as strong disease resistance and high protein content [19]. Numerous resistance genes have been identified in wild emmer, encompassing Powdery mildew resistance: *Pm16*, *Pm30*, *PmG3M*, and *MLIW30* [20,21,22,23]; Rust resistance: *Yr15*, *Yr35*, and *Yr36* [24,25,26]; and Abiotic stress tolerance: salt-responsive *TmHKT1* and *TdCBL6* [27,28] and drought-adaptive *TMPIT1* and *TdAtg8* [29,30]. Beyond its well-characterized resistance profile, wild emmer holds significant potential for grain quality enhancement, as exemplified by the *Gpc-B1* locus, which coordinately upregulates grain protein content and biofortifies essential micronutrients including iron (Fe) and zinc (Zn) [31]. Despite these valuable attributes, most research about wild emmer has focused on gene mapping for desirable traits, with relatively little attention given to its potential for inducing chromosomal structural variations. In this study, we investigated the mechanism causing weak plants in progeny derived from the wild emmer wheat chromosome arm substitution line CASL4AL. Our results clarify wild emmer’s capacity to induce chromosomal variation, providing a useful starting point for further investigations into the underlying processes.

## 2. Results

### 2.1. Some Offspring of (CS x CASL4AL) Grows Weakly

**Figure 1:**
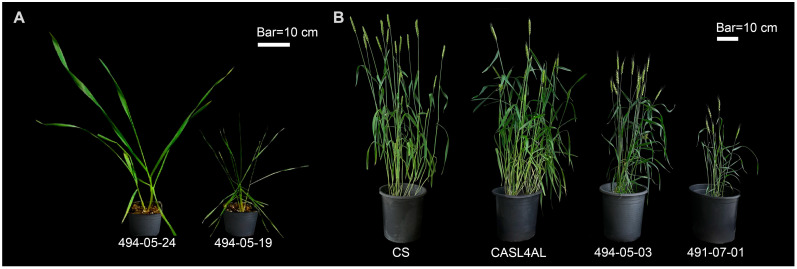
Phenotypic differences between weak plants and their parents (Chinese Spring, CS, and CASL4AL) at seedling (**A**) and maturity (**B**) stage. The plant on the left in (**A**) is 494-05-24 (normal plant), and on the right is 494-05-19 (weak plant). From left to right in (**B**): CS, CASL4AL, 494-05-03 (weak plant), 491-07-01 (weak plant). The scale bar = 10 cm.

**Figure 2:**
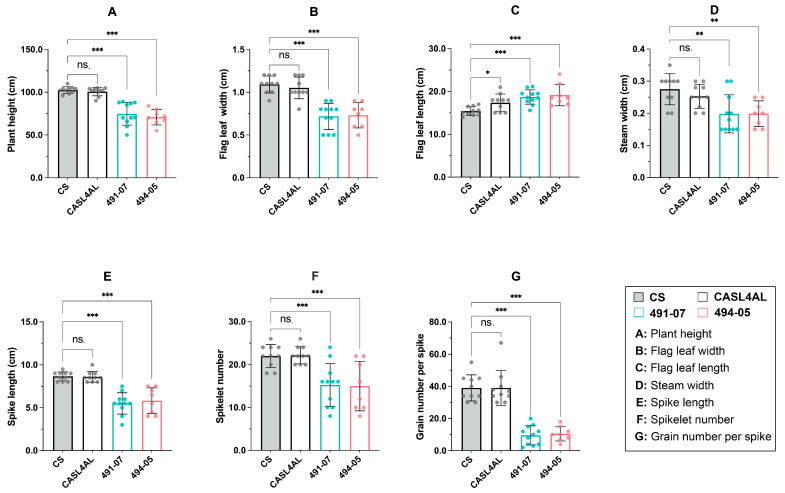
Agronomic traits (**A**–**G**) of weak plants from 491-07 and 494-05 lines and their parents. The *x*-axis represents the plants, the *y*-axis represents the measured values of agronomic traits. *, **, *** represent significance at *p*-values ≤ 0.05, 0.01 and 0.001, respectively. ns. represents no significance at *p*-values > 0.05. Analysis of variance (ANOVA) and the least significant difference (LSD) were used to test the significance.

Analysis of agronomic traits revealed that among a subset of 491-07 and 494-05 F_3:4_ progeny, with crosses between wild emmer wheat chromosome arm substitution line CASL4AL and Chinese Spring (CS) cultivar, arose spontaneously stunted growth (Figure 1). Further examination displayed a significant decrease (*p* < 0.05) from CS in terms of plant height, flag leaf width, spike length, spikelet number, and grains per spike (Figure 2, Table S1). The most severe reductions occurred in flag leaf width, with mean data showing a 34% and 30% decrease in 491-07 and 494-05 lines, respectively, and a reduction in grain number per spike of 75% and 64%. In certain extreme instances, the grain number per spike plummeted to two grains, highlighting the severe spike sterility that significantly constrains offspring production in these weakened lines.

### 2.2. RNA-seq Analysis on Each Chromosome

**Figure 3:**
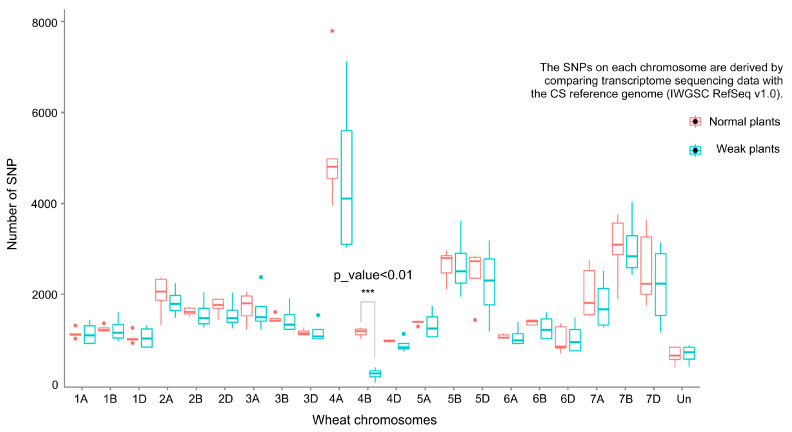
SNP counts on each chromosome based on transcriptome sequencing data of weak and normal plants, respectively. The *x*-axis represents wheat chromosomes, the *y*-axis represents the average number of SNP. The red box type represents the normal plants’ data, and the green box type represents the weak plants’ data. *** indicates an extremely significant difference (*p* ≤ 0.001). The SNP counts on chromosome 4B showed an extremely significant reduction in weak plants as compared with normal plants.

To elucidate the molecular mechanisms underlying weak seedling development, we conducted transcriptome sequencing (RNA-seq) and bulked segregant RNA-seq (BSR-Seq) analysis on weak and normal plants of 491-07 and 494-05 lines. Combining data from 6 RNA-seq (3 weak plants and 3 normal plants) and 4 BSR-seq (2 weak bulk and 2 normal bulk) SNP detections, we found an average of 303 and 1215 SNP counts on chromosome 4B in weak and normal samples, respectively, showing a significant reduction (*p* < 0.001). Notably, no significant differences in SNP counts were observed on other chromosomes between weak and normal plants (Figure 3).

**Figure 4:**
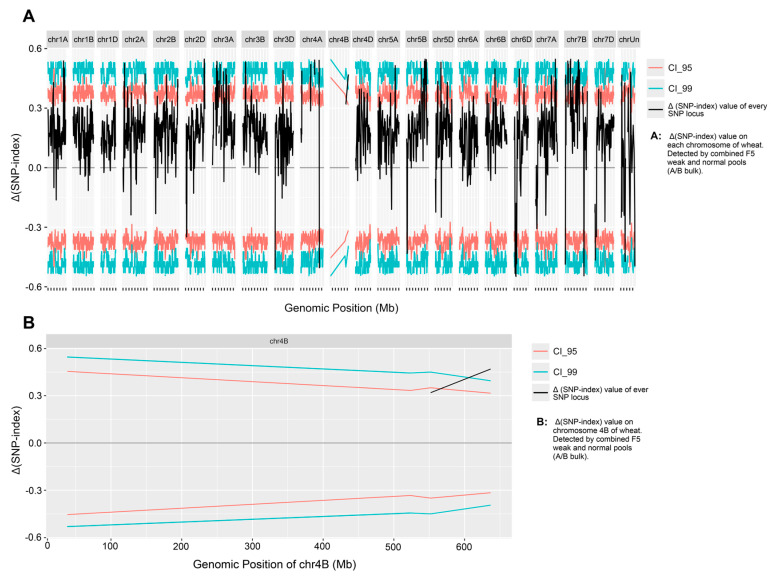
Δ(SNP-index) values on each chromosome (**A**) by BSR-seq of combined weak and normal pools (A and B bulk), and the Δ(SNP-index) values on chromosome 4B (**B**). The Δ(SNP-index) = (SNP-index of the B bulk) − (SNP-index of the A bulk). The *x*-axis represents wheat chromosomes’ genomic position; the *y*-axis represents the Δ(SNP-index). The black lines represent the Δ(SNP-index) value of every SNP locus. Magenta lines (CI_95) mean 95% confidence interval, and turquoise lines (CI_99) mean 99% confidence interval. Only 271 SNPs were detected on chromosome 4B, causing most Δ(SNP-index) values on 4B to be absent.

BSR-seq analysis corroborated this finding. When using BSR-seq results for weak trait-control gene association, in the F_5_ generation pools (A-bulk: weak; B-bulk: normal), chromosome 4B exhibited only 271 detectable SNPs in the weak pool, while other chromosomes maintained > 1000 SNPs each, with the substituted 4A chromosome arm reaching 6000 SNPs (Figure 4), causing most Δ(SNP-index) values on 4B to be absent. This depletion pattern was replicated in the F_4_ generation pools (E-bulk: weak; F-bulk: normal) (Figure S1).

**Figure 5:**
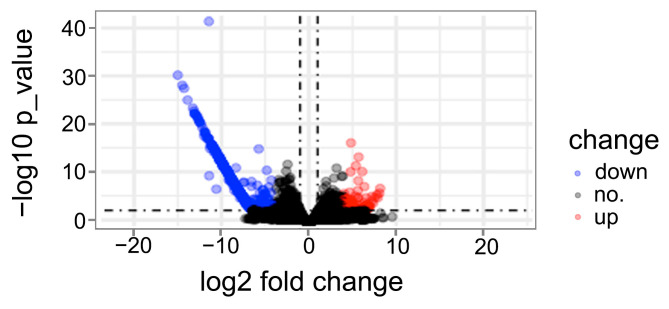
Volcano plot comparing the DEGs between weak and normal plants from RNA-seq data. The *p*-values < 0.05 of log2 fold change ≥ 1 and log2 fold change ≤ −1 are in red and blue dots, respectively, showing the significant upregulated and downregulated genes. Black dots indicate the remaining genes present in the array that were not significantly changed. The values of log2 fold change < −10 concentrated on chr4B.

**Figure 6:**
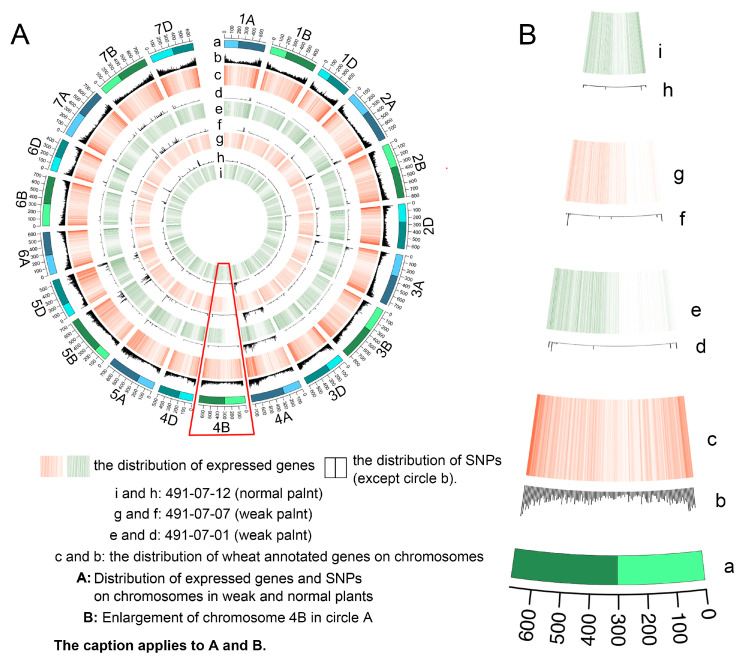
Distribution of expressed genes and SNPs on chromosomes in weak and normal plants (**A**) and enlarged image of chromosome 4B (**B**). a: the length of chromosome and the position of centromere in wheat. The light color is the short arm of the chromosome and the dark color is the long arm of the chromosome. c and b: the distribution of wheat annotated genes on chromosomes. e and d: the distribution of 491-07-01’s (weak plant) detected SNPs and expressed genes; g and f: the distribution of 491-07-07’s (weak plant) detected SNPs and expressed genes; i and h: the distribution of 491-07-12’s (normal plant) detected SNPs and expressed genes. The distribution of expressed genes and SNPs on the short arm of chromosome 4B in weak plants is shown in the red box.

We identified differentially expressed genes (DEGs) between phenotypically weak and normal plants using RNA-seq data. Gene expression levels were quantified via reference-aligned read counts, with DEGs defined using stringent thresholds (|log2 fold change| ≥ 1.0, *p* < 0.05). Differential expression patterns were visualized in a volcano plot (Figure 5). Strikingly, extreme downregulation events (log2 fold change ≤ −10) showed strong chromosomal bias, predominantly localized to chromosome 4B. Chromosomal expression profiling revealed substantial transcriptional suppression in weak plants (Figure 6). Three key observations emerged: 1. The short arm of 4B exhibited markedly reduced gene representation in weak plants compared to normal counterparts and the CS reference genome (evidenced by faded hybridization bands); 2. Total 4B-aligned reads decreased significantly in weak plants (1701 reads, *p* < 0.01) versus normal plants (2597 reads) and the reference (2896 reads); 3. Regional bias: Segmental decomposition identified a critical 0–203 Mb interval accounting for 76% of total read loss (679/896 missing reads), while the distal 471 Mb region (203–674 Mb) contributed only 217 read reductions (Table 1).

**Figure 7:**
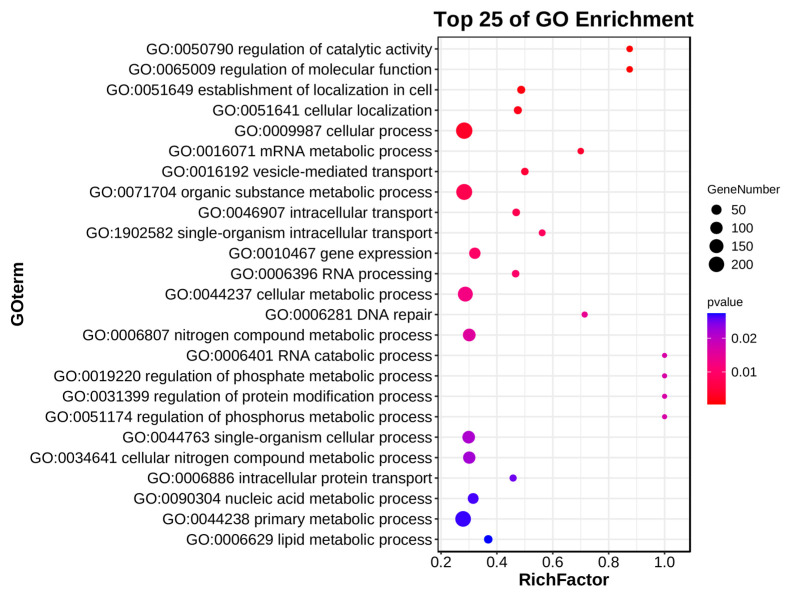
GO enrichment analysis of differentially expressed genes on chromosome 4B in the top 25 GO terms. Each point in the graph represents a GO term. The size of the spot represents the number of differentially expressed genes enriched in the term. The point in the graph represents the significance of enrichment (*p* < 0.05).

**Figure 8:**
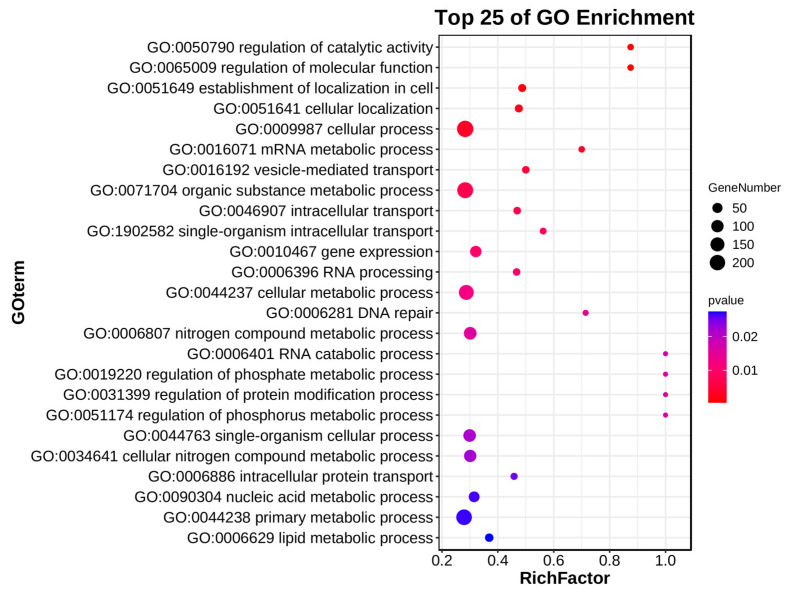
KEGG functional enrichment analysis of differentially expressed genes on chromosome 4B in the top 25 pathways. Each point in the graph represents a pathway. The size of the spot represents the number of differentially expressed genes enriched in the pathway. Only the mRNA surveillance pathway is significantly enriched (*p* < 0.05).

For the purpose of exploring the biological functions and signaling pathways associated with the DEGs on chromosome 4B, GO (Gene Ontology) enrichment and KEGG (Kyoto Encyclopedia of Genes and Genomes) pathway analyses were performed on 1244 downregulated DEGs. Among the top 25 enriched terms (*p* < 0.05), the main significantly associated biological processes included regulation of catalytic activity and molecular function (GO:0050790/GO:0065009), RNA metabolic processes (GO:0016071/GO:0006396/GO:0006401), transport systems (GO:0016192/GO:0046907/GO:1902582), and DNA repair (GO:0006281) (Figure 7). As for the KEGG pathway analysis results, only the mRNA surveillance pathway was significantly enriched (*p* < 0.05). Although other pathways did not reach statistical significance, the ribosome, linoleic acid metabolism, and spliceosome pathways contained relatively high gene numbers with rich factors > 0.3 (Figure 8).

On the other hand, we utilized the WheatOmics [32] website (http://wheatomics.sdau.edu.cn/, accessed on 12 December 2023) to identify potential candidate genes that might be deleted in the weak plants. Functional analysis revealed that the identified 4B genes are associated with critical agronomic traits, including plant height, pollen fertility, inflorescence structure, heading date, root morphology, and stress resistance (Table S2). Of particular interest, *Ms1* (located at 13.125–13.127 Mb) [33], a gene essential for pollen development, falls within the 0–200 Mb region. Silencing of *Ms1* results in severe pollen sterility, a phenotype consistent with the reproductive defects observed in weak plants. Furthermore, *TaMOR* (positioned at 605.691–605.693 Mb) [34], a homolog of the rice *OsMOR* gene, is known to positively regulate plant height, spike length, and grain number when overexpressed. Loss or silencing of *TaMOR* may result in overall weak growth of the plant. These findings indicate that these genes may be deleted in weak plants.

### 2.3. Identification of Chromosome Fragment Deletions Using SSR Maker Analysis

**Figure 9:**
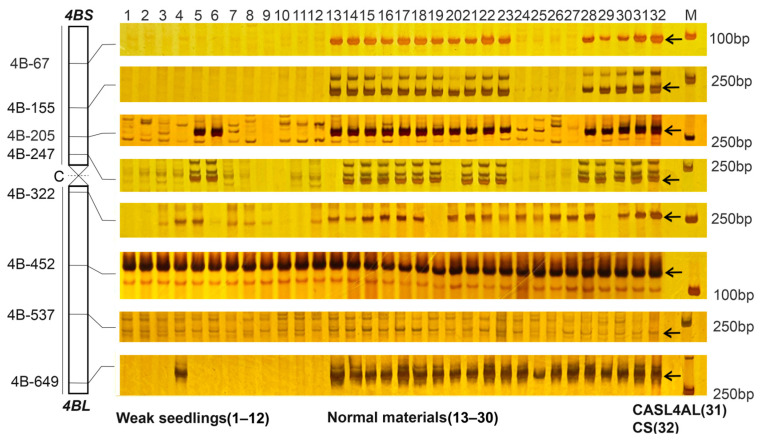
PCR profiling of SSR markers in weak and normal plants from 491-07 and 494-05 lines and their parents. The numbering of the SSR markers and their distributions on chromosome 4B are shown on the left, and the makers are named by chromosome-amplification region, the regions are in Mb. M: molecular marker. Lane 1–12: weak plants. Lane 13–30: normal plants. Lane 31: CASL4AL. Lane 32: CS. The arrow indicates the chromosome 4B specific bands.

To verify structural variations on chromosome 4B in weak plants, we performed molecular marker screening using 4B-specific SSR markers (primer sequences in Table S3). The analysis confirmed a segmental deletion in this chromosome, with a complete absence of amplification in weak plants for markers 4B-67 and 4B-155. Additionally, 10 plants did not amplify at 4B-247 and 11 did not amplify at 4B-649 (Figure 9). In stark contrast, amplification was successful across all markers in the parental and most normal plants. Pearson’s correlation analysis showed a highly significant association (*p* < 0.01) between the weak phenotype and the number of amplification bands, indicating that the observed deletion on chromosome 4B is a crucial factor contributing to the weak phenotype.

Subsequently, thirty-eight F_4:5_ progeny derived from the weak plants, 491-07-05 (n = 8) and 494-05-03 (n = 30), were cultivated continuously during winter 2022 for agronomic trait evaluation and 4B structural analysis. Among these progeny, 27 exhibited no significant differences in any of the observed agronomic traits compared to the control strain (CS), while the remaining 11 displayed weak growth in at least one agronomic trait (Table 2). SSR marker analysis identified chromosomal deletions in 21 progeny, including all weak-growth individuals. Notably, it was discovered that eight offspring had developed novel chromosomal segmental deletions. While F_4_ weak plants 491-07-05 and 494-05-03 showed amplification at loci 4B-247 and 4B-452, respectively, in their progeny, three plants from 491-07-05 and five plants from 494-05-03 failed to amplify the target bands at these loci. These findings demonstrate segregation in phenotypes and chromosome deletions, revealing genetic instability in these deletion lines.

## 3. Discussion

Wheat–wild relative germplasm represents one of the most widely utilized genetic resources for modern wheat improvement. Scientists primarily employ translocation lines to introduce beneficial genes from wild relatives into common wheat [35,36,37]. Among the induction methods of translocation lines, gametocidal (GC) chromosomes and radiation-induced approaches yield the highest translocation frequencies while maintaining technical simplicity [38,39,40]. However, the random nature of chromosomal breakpoints and rejoining sites in these methods often produce materials with trait defects and genome instability, requiring extensive germplasm innovation before being applied to breeding [41]. An alternative approach involves suppressing *Ph1* gene expression to induce homoeologous pairing [42,43,44]. This method creates translocation lines, primarily compensatory translocations, but it still possesses several limitations such as tall plant stature, poor lodging resistance, and low grain yield [45]. Therefore, developing methods that can make smaller alien segment translocations with minimal disruption caused by the presence of the alien segment and the absence of replaced wheat chromatin has been a priority.

In our laboratory, a study conducted in 2015 determined a genetic system on the chromosome arm of wild emmer wheat (*Triticum turgidum* L. var. *dicoccoides*) that interacts with the cytoplasm of Chinese Spring (*Triticum aestivum* L.), leading to chromosome elimination [46]. This study observed chromosomal deletions in the progeny of the wild emmer substitution line, reconfirming its capacity to induce chromosomal variations. As the tetraploid progenitor of common wheat, wild emmer contributes to 2/3 of the genetic material of wheat and provides an indispensable genetic pool for improving wheat productivity and environmental resilience [18,47,48]. Its high genomic similarity to common wheat facilitates hybridization and the formation of genetically stable compensatory translocations. We believe that if wild emmer-induced chromosomal variation mechanisms are confirmed, wheat breeding will be significantly advanced.

In our study, chromosome 4B deletions resulted in narrower leaves, weaker stems, and decreased plant height, all of which contributed to the overall poor growth. Similar phenotypic effects from substantial 4B deletions, such as low fecundity and thin stems and leaves, were observed by Miraghazadeh et al. [49]. Endo et al. documented gametophytic sterility following 4B short-arm deletions induced by GC chromosomes [15]. These consistent findings suggest that 4B harbors critical growth-regulating factors. Among the characterized 4B genes, most control plant height, fertility, inflorescence architecture, heading date, root morphology, and stress responses (Table S2). Notably, *TaMOR*, which is essential for root morphogenesis and vegetative growth [34], could severely impair root development and nutrient uptake when deleted. Similarly, suppression of the *Ms1* gene (around 10 Mb on Chr 4B) [33] causes widespread pollen sterility. Our gene functional enrichment analysis revealed that most of the deleted genes on chromosome 4B are primarily involved in RNA metabolic pathways, including RNA processing, metabolism, and gene expression regulation. Accumulating evidence indicates that mRNA stability is essential for precise post-transcriptional regulation [50], while non-coding RNAs (ncRNAs) are important regulators of gene expression involved in various metabolisms [51], long non-coding RNAs (lncRNAs) play pivotal roles in pollen and anther development [52]. In addition to the RNA metabolic pathways, DNA repair and metabolism of compounds in the cell were also significantly enriched in our analysis. Collectively, these results demonstrate that chromosome 4B harbors a functionally coherent gene network governing pollen development, overall plant growth vigor, and DNA genetic stability, and these genes may exhibit strong gene dosage effects in wheat, as the substantial deletion of genes on chromosome 4B likely leads to their downregulation, thereby impairing normal growth. Further functional characterization of the identified DEGs on chromosome 4B will be one of the focuses of future research.

Transcriptome sequencing and molecular marker analysis are used to identify chromosomal fragment deletions in this study. Conventional methods like in situ hybridization (ISH) coupled with marker analysis allow direct visualization of chromosomal changes and precise breakpoint mapping [12,16,53]. For instance, chromosome 5P of *Agropyron cristatum* was found to be able to induce translocations and deletions by employing these methods [16]. However, ISH requires extensive expertise and technical proficiency, which can pose challenges for laboratories with limited resources [54]. Next-generation sequencing (NGS) technologies like transcriptome and whole-genome sequencing now provide high-precision, low-input alternatives for detecting chromosomal variations For example, genome sequencing was used in the experiments of Li et al. and Zhai et al. to precisely map introgression boundaries and identify terminal deletion genes on chromosome 4A [55,56]. In our study, ISH was inapplicable, because the DNA of wild emmer wheat bears a strong resemblance to that of common wheat, making it challenging to differentiate between them using wild emmer wheat’s DNA as a probe. Currently, our laboratory is devoid of this technology. However, we found that RNA sequencing analysis can efficiently distinguish the chromosomal DNA of wild emmer wheat from the chromosomal composition of common wheat [57]. Thus, we adopted transcriptome sequencing as the primary detection method, supplemented by marker analysis to characterize 4B deletions. This integrated pipeline offers a robust, accessible solution for chromosomal variation studies in resource-constrained settings.

## 4. Materials and Methods

### 4.1. Plant Materials and Field Trials

The plant materials used were Chinese Spring wheat (CS, *Triticum aestivum* L., 2n = 6x = 42, AABBDD), chromosome arm substitution line CASL4AL, and their F_3:4_, F_4:5_ lines, 491-07 and 494-05. CASL4AL was developed by introgressing the 4AL arm from wild emmer wheat (*Triticum turgidum* L. var. *dicoccoides*, 2n = 4x = 28, AABB) accession TTD 140 into common wheat CS, provided by Professor Moshe Feldman in Israel [58]. The breeding strategy was detailed by Lu et al. [57]. Progeny from the CASL4AL × CS cross was assigned numerical identifiers from the F_2_ generation onward. Each subsequent generation was designated by appending “- [plant number]” to the parental line code (e.g., 491-07 represents an F_3_ plant derived from F_2_ line 491).

Seeds were germinated in seed germination boxes under the condition of 26 °C with a 16-h light/8-h dark photoperiod cycle during the winter of 2021 and 2022. Uniform seedlings were selected and transferred into pots (diameter: 25 cm, height: 40 cm) when their second true leaf reached 2–4 cm in length, with three seedlings planted per pot. The planting soil consisted of a 1:1 mixture of seedling substrate (peat: vermiculite: perlite = 1:1:1) and paddy soil. All seedlings planted in pots were subject to unified cultivation and management in the greenhouse (26 °C, nature sunshine) of the agricultural garden at Zhejiang A&F University in Lin’an (30°23′ N, 119°43′ E, 44.7 m above sea level), Zhejiang, China, with applications of a nitrogen–phosphorus–potassium(N/P/K) compound fertilizer and timely water management to ensure optimal growth.

### 4.2. Agronomic Trait Measurement

Agronomic trait quantification was performed on F_3:4_ progeny of lines 491-07 and 494-05 in 2021, and all progeny produced by weak plants in 2022. In each round, 10 randomly selected plants from CASL4AL and CS accessions would be measured as parental reference data. Measurements were conducted at two developmental stages: heading stage (defined as 50% emergence of mature spikes) and physiological maturity (characterized by complete yellowing of all tillers). The following parameters were recorded according to standardized protocols:

At heading stage:

Flag leaf length (FLL): Distance from ligule to leaf tip on the main spike;

Flag leaf width (FLW): Maximum width at the mid-point of flag leaf blade;

Stem width (SW): Diameter measured 1 cm below the penultimate node of main culm.

At maturity:

Plant height (PH): Vertical distance from soil surface to spike apex (excluding roots and awns);

Spike length (SL): Length of main spike from base to tip (awns excluded);

Spikelet number (SLN): Total spikelets on primary spike;

Grain number per spike (GPS): Fully developed grains on primary spike.

### 4.3. RNA-seq Analysis

#### 4.3.1. Sample Preparation and RNA Isolation

Fourth true leaves (15–16 cm in length) from 491-07 and 494-05 progeny were collected at 10:00 AM. Samples were wrapped in sterile aluminum foil, labeled, flash-frozen in liquid nitrogen within 5 min of collection, and stored at −80 °C until further processing. Phenotype-based sample grouping was implemented as follows:

RNA-seq Analysis: A total of 6 samples were sequenced, including 3 weak samples and 3 normal samples from F_4_ generation of 491-07, with each sample containing an individual plant.

BSR-seq Analysis: Four bulks (A, B, E, and F) were sequenced, with each bulk consisting of 8 individual plants.

A-bulk: Formed by three weak F₅ plants from line 491-07-05 and five weak F₅ plants from line 491-07-01.

B-bulk: Comprised of eight normal F₅ plants from line 491-07-12.

E- and F-bulks: Eight weak and eight normal F₄ plants from line 494-05 were used to form E-bulk and F-bulk, respectively.

Total RNA was isolated using the RNAprep Pure Plant Kit (DP190102, TianGen Biotech, Beijing, China) following manufacturer protocols. RNA integrity was verified through 1% agarose gel electrophoresis using a GelDoc XR+ imaging system (Bio-Rad, Hercules, CA, USA), with distinct 28S and 18S ribosomal bands serving as quality indicators. RNA quantification was performed using a NanoDrop^TM^ 2000 spectrophotometer (Thermo Scientific, Wilmington, DE, USA), with absorbance ratios (A260/A280) maintained between 1.8 and 2.0 for all samples.

#### 4.3.2. cDNA Library Preparation and Sequencing

RNA-seq library construction and sequencing were conducted by Novogene Bioinformatics Technology Co., Ltd. (Beijing, China) following Illumina standardized protocols. Using oligo(dT) magnetic beads to enrich mRNA, and adding segmental buffer broke the mRNA into short fragments and reverse-transcribed it into double-stranded cDNA. Additionally, cDNA libraries were prepared with the KAPA HyperPrep Kit (KAPA Biosystems, Wilmington, MA, USA), with fragment size distribution verified on an Agilent 2100 Bioanalyzer (Agilent Technologies Inc., Santa Clara, CA, USA) and quantified via qPCR. Sequencing was performed on an Illumina HiSeq 2000 system (Illumina, San Diego, CA, USA) with 150-bp paired-end reads (12 Gb depth per sample).

The raw sequence data in this paper have been deposited in the Genome Sequence Archive in the National Genomics Data Center, and are publicly accessible at https://ngdc.cncb.ac.cn/gsa (accessed on 27 March 2025) [59,60]. The accession number is CRA024112.

#### 4.3.3. Alignment of RNA-seq Reads and SNP Analysis

RNA-seq raw data processing was performed on a Linux system through the following pipeline: Raw sequences were quality-controlled by removing adapters and trimming reads containing > 50% low-quality bases (Q ≤ 20) using fastp v0.19.5 [61]. The high-quality clean reads were then aligned to the CS reference genome (IWGSC RefSeq v1.0) [4] using Hisat2 [62]. Unique alignments were extracted, with SAM files converted to sorted BAM files using Samtools v1.9 [63], followed by PCR duplicate marking via GATK4.0’s MarkDuplicates module [64]. Variant calling was performed using GATK’s HaplotypeCaller, with SNPs filtered using stringent thresholds: “QUAL < 60.0, QD < 2.0, MQ < 40.0, FS > 60, SOR > 3.0, DP < 10, MQRankSum < −12.5, ReadPosRankSum < −8.0”. This workflow was applied uniformly to all samples for SNP calling. Pure SNP counts (1/1) and their distribution positions were detected by Microsoft Excel 2013, with selected data visualized based on specific analytical requirements.

For BSR-seq analysis, the Δ(SNP-index) was calculated using the QTLseqr package, developed by Mansfeld et al. in 2018, a specialized tool for next-generation sequencing-based QTL mapping [65].

#### 4.3.4. Identification and Annotation of Differentially Expressed Genes

Differential gene expression analysis was performed using featureCounts (Rsubread package v2.0.1) [66] in R studio v3.6.1 to quantify mapped reads and identify DEGs between weak and normal plants, applying thresholds of |log2 fold change| > 1 with a *p*-value ≤ 0.05. Volcano plots were generated to visualize the significant DEGs, from which we isolated 1244 DEGs (all downregulated) located on chromosome 4B for the next analysis. Then, we conducted Gene Ontology (GO) and Kyoto Encyclopedia of Genes and Genomes (KEGG) analyses on these DEGs. Functional annotation was employed using in-house Perl scripts and KofamKOALA (https://www.genome.jp/tools/kofamkoala/, accessed on 26 March 2025) for GO_id and K_id identifier assignment, followed by GO terms and KEGG pathways enrichment analysis via OmicShare tools (https://www.omicshare.com/tools/, accessed on 26 March 2025). A hypergeometric test was employed to define the significance of enrichment analysis for GO categories.

### 4.4. SSR Marker Analysis

#### 4.4.1. SSR Marker Development

SSR loci in the 0–650 Mb region of chromosome 4B were predicted using MISA (MicroSAtellite, http://pgrc.ipk-gatersleben.de/misa/misa.html, accessed on 10 December 2021) with the CS reference genome (IWGSC RefSeq v1.0), applying thresholds of ≥ 6 repeats for dinucleotides and ≥ 5 repeats for triplet, quadruplet, and pentanucleotides. Starting at the 50 Mb position, three primers targeting 4B-specifical amplifying on chromosome 4B were developed at 100 Mb intervals based on the predicted loci. Primers were designed using the wheat public primer design page (http://wheatomics.sdau.edu.cn/PrimerServer/, accessed on 10 December 2021) [32], setting the max product size to 300 bp, and the checking database selected “Chinese_Spring1.0.genome” and “Wild_emmer.Genome”, with other parameters set to default. Primers were synthesized by BGI Biotechnology (Shanghai, China).

#### 4.4.2. Chromosome Structure Analysis

We sampled 3 cm lengths of leaves before plant heading for DNA extraction via the Cetyltrimethylammonium Bromide (CTAB) method. DNA quality was assessed using a NanoDrop™ spectrophotometer (Thermo Scientific, Wilmington, DE, USA), with OD260/280 = 1.8–2.0 and OD260/230 ≥ 2.0 considered acceptable. DNA concentration was adjusted to approximately 150 ng/uL. PCR amplification was conducted following Lu et al. [57]. Amplified products were separated on a 12% polyacrylamide gel stained with ethidium bromide. The seven paired primers used in this study to identify the structure of chromosome 4B were shown on Table S3.

### 4.5. Statistical Analysis and Visualization

Agronomic trait differences between weak plants and CS controls were analyzed using SPSS 19.0. One-way ANOVA with LSD test compared group means. The SNP counts difference on each chromosome of weak and normal plants based on the transcriptome sequencing (RNA-seq and BSR-seq) datasets were similarly assessed by LSD. We used one sample t-test to compare the difference of individual plant agronomic trait value with CS, making CS population data the Test Variable(s) and the individual plant measurements the Test Value. Pearson’s correlation coefficient was calculated between phenotype and presence of target bands at marker loci, designating each weak plants as 0 and normal as 1, and corresponds to the amplified bands number, respectively. Significant differences in the above tests were defined as *p*-values ≤ 0.05.

The column chart for agronomic trait analysis was drawn using GraphPad Prism 10. Figures of SNP counts and distribution of expressed genes were completed by R studio 3.6.1, using packages “circlize” and “ggplot2”. The images of plants and SSR maker analysis were processed by Adobe Photoshop 2019 and Adobe Illustrator 2022.

## 5. Conclusions

In summary, we identified weak plants in the progeny of wild emmer wheat (*Triticum turgidum* L. var. *dicoccoides*) chromosome substitution lines, which showed reduced plant height, thinner stalks, narrower flag leaves, and shorter spike length compared to the parent Chinese Spring (*Triticum aestivum* L.). Segmental deletions on wheat chromosome 4B were detected in weak plants using transcriptome sequencing and SSR molecular marker analysis. The primary missing regions were the 0–247 Mb interval and the terminal region of the long arm on chromosome 4B. Pearson’s correlation coefficient indicates that the deletion on chromosome 4B is the main cause of weak plants. This study reaffirms the potential of wild emmer to induce chromosomal variations. However, further research is needed to determine whether wild emmer contains stable mechanisms (such as the Gc chromosome of *Aegilops*) for creating chromosomal variations.

## Figures and Tables

**Table 1:** Average expressed gene reads on chromosome 4B through six RNA-seq data of weak and normal plants from 491-07.

Chromosome Range (Mb)	CS RefSeqv1.0	Normal Plant Samples	Weak Plant Samples
0–203	1444	998	319
203–674	2425	1599	1382
0–674	3869	2597	1701

**Table 2:** Summary of agronomic traits in offspring of 491-07-05 and 494-05-03 with chromosome deletions.

Plants Number	PH	FLL	FLW	SW	SL	SLN	GPS
491-07-05	↓	↑	↓	↓	↓	↓	↓
491-07-05-01	ns.	ns.	ns.	ns.	ns.	ns.	ns.
491-07-05-02	ns.	ns.	ns.	ns.	ns.	ns.	ns.
491-07-05-03	ns.	ns.	ns.	ns.	ns.	ns.	ns.
491-07-05-04	↓	↑	↓	↓	↓	↓	↓
491-07-05-05	ns.	ns.	ns.	ns.	ns.	ns.	ns.
491-07-05-06	ns.	ns.	ns.	ns.	ns.	ns.	ns.
491-07-05-07	↓	ns.	ns.	↓	ns.	↓	↓
491-07-05-08	ns.	ns.	ns.	ns.	ns.	ns.	ns.
494-05-03	↓	ns.	ns.	↓	↓	↓	↓
494-05-03-01	↓	↓	ns.	↓	↓	↓	↓
494-05-03-02	↓	↓	ns.	↓	↓	↓	↓
494-05-03-04	ns.	ns.	ns.	ns.	ns.	ns.	ns.
494-05-03-07	ns.	ns.	ns.	ns.	ns.	ns.	ns.
494-05-03-11	↓	↑	↓	↓	↓	↓	↓
494-05-03-12	ns.	ns.	ns.	↓	↓	↓	↓
494-05-03-13	↓	↑	↓	↓	↓	↓	↓
494-05-03-14	↓	**↑**	↓	↓	↓	↓	↓
494-05-03-18	↓	**↑**	↓	↓	↓	↓	↓
494-05-03-24	↓	**↑**	↓	↓	↓	↓	↓
494-05-03-25	ns.	ns.	ns.	ns.	ns.	ns.	ns.
494-05-03-27	ns.	ns.	ns.	ns.	ns.	ns.	ns.
494-05-03-28	↓	**↑**	↓	↓	↓	↓	↓

**↑**, ↓ and ns. indicate a significantly higher, lower (*p* ≤ 0.05), and no significance compared with CS, respectively. PH means plant height, FLL means flag leaf length, FLW means flag leaf width, SW means steam width, SL means spike length, SLN means spikelet number, GPS means grain number per spike.

## Data Availability

The raw sequence data in this paper have been deposited in the Genome Sequence Archive (Genomics, Proteomics & Bioinformatics 2021) in National Genomics Data Center (Nucleic Acids Res 2022), which are publicly accessible at https://ngdc.cncb.ac.cn/gsa, accessed on 27 March 2025. The accession number is CRA024112.

## Supplementary Materials

The following supporting information can be downloaded at: https://www.mdpi.com/article/10.3390/plants14071134/s1, Figure S1: Δ(SNP-index) values on each chromosome (C) by BSR-seq of combined weak and normal pools (E and F bulk), and the Δ(SNP-index) values on chromosome 4B (D); Table S1: Agronomic traits of weak plants from 491-07 and 494-05 lines and their parents; Table S2: Candidate missing genes on chromosome 4B of weak plants (partial list); Table S3: Lists of primers for 4B chromosome structure identification.
