# Supplementary material for: A Bread Wheat Line with the Substituted Wild Emmer Chromosome 4A Results in Fragment Deletions of Chromosome 4B and Weak Plants
Source: Plants (Basel). 2025 Apr 5;14(7):1134. doi: 10.3390/plants14071134 (PMC11991261; doi:10.3390/plants14071134)
Supplement: Supplementary file 1 [file plants-14-01134-s001.zip › plants-3534009-supplementary.pdf]

Table S1: Agronomic traits of weak plants from 491-07 and 494-05 lines and their parents.

	Parents						Weak plants					
	CS			CASL4Al			491-07			494-05		
	mean	range	CV%	mean	range	CV%	mean	range	CV%	mean	range	CV%
Plant height (cm)	102.5	96~110	3.63	100.7	90~105	4.64	74.87***	50~89	16.98	70.88***	55~84	11.89
Flag leaf width (cm)	1.05	0.9~1.2	8.66	1.05	0.8~1.2	11.47	0.72***	0.5~0.9	20.41	0.73***	0.5~0.9	19.02
Flag leaf length (cm)	15.4	14.0~17.0	6.41	17.4*	15~21	11.04	18.71***	15.6~21.2	8.54	19.15***	16.7~24	12.27
Steam width (cm)	0.28	0.2~0.35	16.76	0.25	0.2~0.3	17.07	0.198**	0.15~0.30	28.61	0.199**	0.15~0.25	18.90
Spike length (cm)	8.65	8~9	5.11	8.6	8~10	6.78	5.55***	3~8	22.89	5.88***	4~8	25.44
Spikelet number	22	18~26	11.50	22.2	20~26	8.50	15.45***	8~24	29.76	15***	8~22	35.90
Grain number per spike	39.1	30~55	19.66	39.1	30~67	26.42	9.64***	2~20	59.08	10.50***	4~18	39.84

CV: Coefficient of variation. *, **, *** represent significant differences between CS and materials under the same trait, at *p*-values ≤ 0.05, 0.01 and 0.001, respectively. Analysis of variance (ANOVA) and the least significant difference (LSD) were used to test the significance of differences.

Table S2: Candidate missing genes on chromosome 4B of weak plants (partial list).

Name	Position	Traits	DOI of gene mapped
TaBot1-4B	621.074-621.095 Mb	boron toxicity tolerance	10.1126/science.1146853
Rht-B1/Rht1	30.861-30.863 Mb	plant height	10.1038/22307
BGC1	21.937-21.944 Mb	starch synthesis	10.1093/jxb/erz405
TaMOR	605.691-605.693 Mb	root architecture	10.1093/jxb/erw193
Lr67	504.939-504.944 Mb	leaf rust; stripe rust; stem rust; powdery mildew	10.1038/ng.3439
TaMlo-B1	613.157-613.160 Mb	Powdery mildew resistance	10.1111/pbi.12631
Ms1	13.125-13.127 Mb	male sterility	10.1073/pnas.1715570114
TaGRP2	14.388-14.389 Mb	heading date, flowering times	10.1038/ncomms5572
VRN-B2/VRN2	657.515-657.517 Mb	vernalization	10.1007/s10681-015-1589-7
TaPHT1-9-4B	606.816-606.818Mb	Pi uptake and translocation	10.1111/nph.17534
WSOC1-4B	640.289-640.303 Mb	flowering	10.1111/j.1399-3054.2007.00927.x
TaSRL1-4B	585.821-585.823 Mb	root length; plant height	10.1093/jxb/erab357
TaPRR73-4B	427.490-427.496 Mb	heading date and plant height	10.3389/fpls.2016.00772
SVP3-4B	589.180-589.195 Mb	wheat spike and spikelet development/flowering/plant height	10.1101/2020.12.01.405779
SEP1-2-4B	507.964-507.979 Mb	floral organ differentiation	10.1111/nph.16122
TaNAC071-4B	103.054-103.056 Mb	drought tolerance	10.1016/j.molp.2021.11.007
TaTB1-4B	30.362-30.363 Mb	inflorescence architecture	10.1105/tpc.17.00961

TaDIR-B1	664.392-664.393 Mb	fusarium crown rot	10.1111/pbi.13554
TaDWF4-4B	487.768-487.774 Mb	BR synthesis pathway; carbon assimilation; wheat productivity; N use efficiency	10.1038/s42003-022-03139-9
TaFROG-4B	115.549-115.55 Mb	fusarium head blight resistance	10.1104/pp.15.01056
TaMPK3-4B	426.767-426.769 Mb	ABA; Drought tolerance	10.1111/nph.18326

Table S3. Lists of primers for 4B chromosome structure identification

**Primer name**	**Forward primer (5'-3')**	**Reverse primer (3'-5')**	**Amplification Region/bp**	**Tm value/°C**	**Product Size/bp**
4B-67	AATCATCACCGCATCAAACA	CTAGAGTGTCGTGCGGGC	67,497,440-67,497,541	55	102
4B-155	TTGATACGTGCTTGCAATCTG	TAACTGACGAACAGGGCACA	155,761,501-155,761,729	58	229
4B-247	CTCTTCGAGGAAAACCAACG	AGGATGCTCAATGCTTGCTT	247,923,442-247,923,681	58	240
4B-322	ACTACAAATCCCATGCTGCC	GAGATGCTGGATTGGAGGAG	322,967,804-322,967,912	58	109
4B-452	CTGATGCTTTGTTCCCCAAT	GGTCCTGCACACTTGGAGAT	452,222,217-452,222,336	55	120
4B-537	GAGAGAGAGGAAACCAGGGG	AGAACTAGAGGGAGACGGGG	537,870,344-537,870,579	58	236
4B-649	TGATTGCTCCACTGTCCTGA	ACGTTGCCTCGCATAGAAAG	649,225,442-649,225,664	58	223

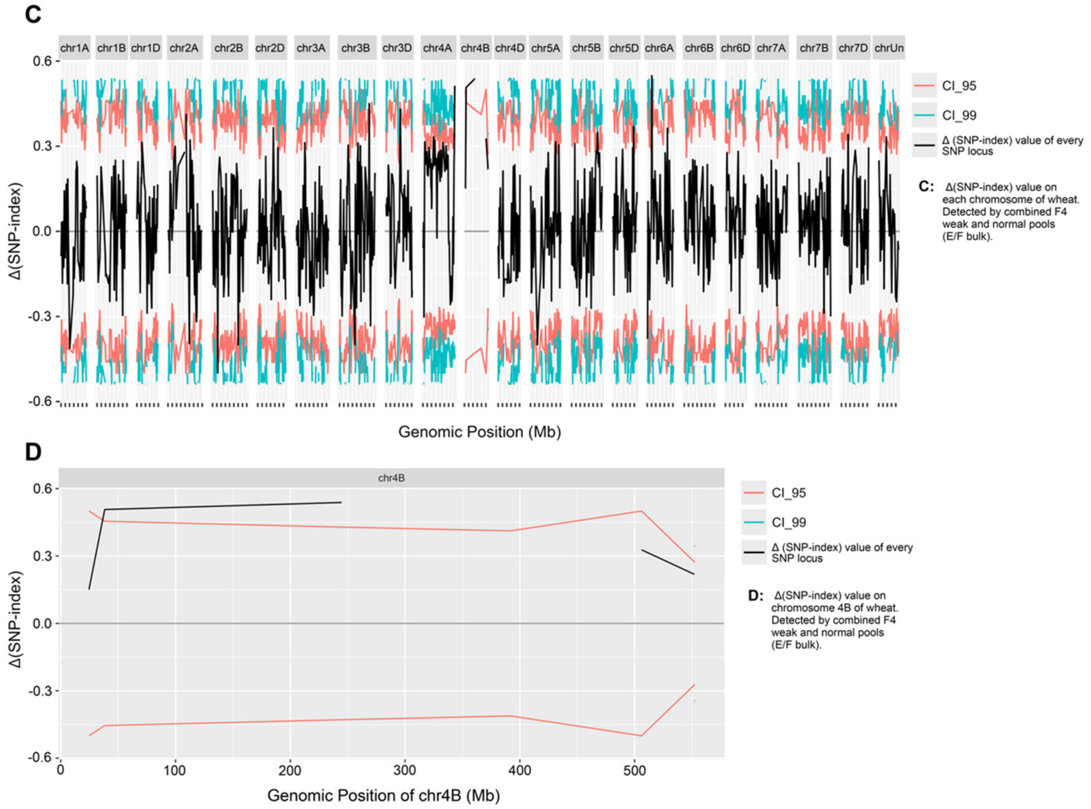

Figure S1: Δ(SNP-index) values on each chromosome (C) by BSR-seq of combined weak and normal pools (E and F bulk), and the Δ(SNP-index) values on chromosome 4B (D). The Δ(SNP-index) = (SNP-index of the F bulk) − (SNP-index of the E bulk). The x-axis represents wheat chromosomes' genomic position; the y-axis represents the Δ (SNP-index). The black lines represent the Δ (SNP-index) value of every SNP locus. Magenta lines (CI_95) mean 95% confidence interval, and turquoise lines (CI_99) mean 99% confidence interval.
